# Gamma-Aminobutyric Acid-Producing *Levilactobacillus brevis* Strains as Probiotics in Litchi Juice Fermentation

**DOI:** 10.3390/foods12020302

**Published:** 2023-01-08

**Authors:** Yiwen Jin, Jinyong Wu, Dan Hu, Jun Li, Weiwei Zhu, Lixia Yuan, Xiangsong Chen, Jianming Yao

**Affiliations:** 1 Institute of Plasma Physics, Hefei Institutes of Physical Science, Chinese Academy of Sciences, Hefei 230031, China; 2 Science Island Branch, Graduate School of USTC, Hefei 230026, China; 3 Hefei CAS Health Bio-Industrial Technology Co., Ltd., Hefei 230031, China; 4 Wuhan Zhongke Optics Valley Green Biotechnology Co., Ltd., Wuhan 430075, China

**Keywords:** *Levilactobacillus brevis*, litchi juice, fermentation, gamma-amino butyric acid, probiotics

## Abstract

*Levilactobacillus brevis* strains can be isolated from traditional Chinese pickles and used as the starter cultures to improve the nutritional profiles of fermented juices. Three *L. brevis* strains (LBG-29, LBG-24, LBD–14) that produce high levels of gamma-aminobutyric acid (GABA; >300 mg/L) were isolated from traditional Chinese pickles. The strains showed tolerance to low pH and high bile salts and exhibited safety in vitro. Litchi juice was fermented using each strain at 37 °C for 48 h. The litchi juice was determined to be a good substrate for fermentation as the process enhanced its functional profile. Overall, cell vitality increased (above 8.7 log_10_ CFU/mL), the antioxidant activities of 2,2-diphenyl-1-picrylhydrazyl (DPPH) and ferric ion-reducing antioxidant power (FRAP) were significantly increased, and the antioxidant capacity of the 2,2′-amino-di(3-ethyl-benzothiazoline sulphonic acid-6)ammonium salt (ABTS) was decreased. There was also a significant increase in the GABA and acetic acid content after LBG-29 and LBG-24 fermentation. It was thus determined that the LBG-29 and LBG-24 strains could be used to improve beverage functionality and aid in the development of new products. This is the first report of litchi fermentation using *L. brevis* as a starter culture. Further research is required to elucidate the functional benefits for the human body and the nutritional and functional properties during its shelf life.

## 1. Introduction

Gamma-aminobutyric acid (GABA) is an important four-carbon free non-protein amino acid produced by glutamic acid decarboxylase through irreversible α-decarboxylation [1]. It is widely distributed among microorganisms, plants, and animals and has a variety of physiological functions [2,3]. Specifically, it helps plants to withstand environmental changes [4], including drought resistance [5], while in mammals, it is the major inhibitory neurotransmitter in the mature central nervous system and is present in high concentrations in different regions of the brain [6]. It aids in the prevention and treatment of multiple diseases, including diabetes [7], depression, anxiety [8], chronic insomnia [9,10], and Alzheimer’s [11]. It is also essential for neuronal development in the early brain [12]. GABAergic signalling is a key component of the brain–gut axis, as the gut microbiota modulate host responses and influence host behaviour by producing active compounds such as GABA [13].

Chemical synthetic GABA usually contains toxic by-products and consequently has poor safety levels and is thus a threat to human health and banned from food use [14]. GABA produced by microbial fermentation, however, is considered relatively safe, particularly when produced by lactic acid bacteria (LAB), which are often used as starter cultures in fermented foods such as pickles, soy milk, and black raspberry juice [15]. Widely used LAB include *Pediococcus pentosaceus* [16], *Lactobacillus paracasei* [17], and *Levilactobacillus brevis* [18], among which *L. brevis* produces the most GABA [19]. Fermented foods rich in GABA are expected to increase in popularity, as supplemental GABA intake from these foods is beneficial to the host at certain doses [20]. There are several recent examples of the incorporation of *L. brevis* in fermented foods, including green tea [21], milk [22], and juice [23]. Such foods are reported to have multiple health benefits such as the regulation of intestinal flora, which can help to improve sleep quality [24] and relieve anxiety [22].

Litchi (*Litchi chinensis* Sonn.) is a member of the Sapindaceae family and an important cash crop that is widely cultivated in subtropical regions [25]. China is a major producer of litchi as its annual output is more than 2 million tons, accounting for 50% of the global output. The direct output value of litchi planting alone is CNY 28 billion a year in China [26]. Litchi is popular because of its sweet taste and nutritive value; its pulp contains multiple beneficial substances, including polysaccharides, vitamins, and minerals that are beneficial to the liver, brain, and heart [27,28]. However, litchi is seasonal, and when freshly picked, it has a short shelf life and spoils easily due to its high moisture content [29,30,31]. Further processing, such as producing liquor and fermented juice, extends the shelf life to better meet the market demand [32,33]. Although many studies have shown that fermentation with different LAB strains has beneficial effects on litchi juice, studies on how to improve the quality of litchi juice using *L. brevis* have not yet been reported.

Considering the above points, we have hypothesised that *L. brevis* fermentation could improve the functional properties and nutritional value of litchi juice. The objective of the present study was to investigate the effects of *L. brevis* fermentation on the physicochemical properties of litchi juice, including its antioxidant capacity and bioactive components. The results of this study will provide new data that will aid in the development of functional foods that utilise *L. brevis* as a starter culture.

## 2. Materials and Methods

### 2.1. Strain Isolation and Identification

#### 2.1.1. Isolation

Pickled cucumber, bamboo shoots, bell pepper, and white radish were fermented for 4 d, and the resulting juices were used for LAB isolation. Sterile glycerol was then added to a final concentration of 30%, and each juice type was stored at −20 °C until further analysis. The LAB samples were screened using semi-selective de Man, Rogosa, and Sharpe (MRS) agar medium consisting of 1 mL/L Tween-80, 20 g/L glucose, 5 g/L NaAc•3H_2_O, 2 g/L C_6_H_5_O_7_(NH_4_)_3_, 2 g/L K_2_H_P_O_4_•7H_2_O, 10 g/L tryptone, 5 g/L beef extract powder, 4 g/L yeast extract, 0.2 g/L MgSO_4_•7H_2_O, 0.05 g/L MnSO_4_•4H_2_O, and 15 g/L agar powder. Sterile normal saline was used to serially dilute each isolate 10-fold. Fifty microlitres of each dilution was then inoculated onto MRS agar and incubated at 37 °C for 48 h. Single colonies were then selected for purification. Each purified strain was then dripped with 10% H_2_O_2_ to observe the formation of air bubbles, and Gram staining was performed. The catalase-negative and Gram-positive purified strains were further analysed and activated three times before each use.

#### 2.1.2. Species Identification

Bacteria were collected by scraping single colonies from the MRS agar. Then, 16S rRNA sequencing was performed by General Biosystems (Chuzhou, China), and the identified sequences were compared using the NCBI website (https://blast.ncbi.nlm.nih.gov/Blast.cgi (accessed on 21 October 2021)). The strains used in this study are listed in Table 1.

#### 2.1.3. Strain Screening

Each LAB was inoculated into the MRS fermentation medium containing 1% (*w*/*v*) monosodium glutamate (1% MSG-MRS) and incubated for 48 h at 37 °C. Bacteria were pelleted at 2500× *g* for 10 min at 18 °C. The GABA in the supernatant was then measured as described in Section 2.1.4.

#### 2.1.4. GABA Measurement

Assays were performed as previously described [34,35] with slight modifications, using an Essentia LC-16 high-performance liquid chromatography (HPLC) system (Shimadzu, Kyoto, Japan) equipped with an auto-sampler, binary gradient pump, and UV detector. Samples required derivatisation before HPLC detection. Sample supernatants (100 μL) were mixed with 100 μL 0.5 M NaHCO_3_ and 200 μL dansyl chloride acetone solution (8 g/L) and then shaken. The mixture was then derivated at 30 °C for 1 h in the dark. Dansyl chloride derivatives were separated using WondaSil C18-WR (4.6 mm × 250 mm, 5 μm) and detected by absorbance at 254 nm. Filtration was performed using a 0.45 μm filter membrane, and the injection volume was 20 μL. Separation was accomplished using a gradient program, which was as follows: 0 min, 20% A; 3 min, 20% A; 18 min, 50% A; 22 min, 100% A; 24 min, 100% A; 25 min, 20% A; and 30 min, 20% A, where mobile phase A was methanol and B was a 5:75:420 (*v*/*v*) mixture of tetrahydrofuran, methanol, and 0.05 M sodium acetate solution (pH 6.2), respectively. The flow rate was set to 1 mL/min, and the column was maintained at 25 °C (R^2^ = 0.9998).

### 2.2. Strain Characterisation

#### 2.2.1. Physio-Biochemical Assays

Methyl red, indole, and nitrate reduction assays were performed as previously described [36]. V-P, hydrolysed hippurate, starch hydrolysis, gelatin liquefaction, urease production, esculin hydrolysis, and hydrogen sulphide production assays were also performed as previously described [37].

#### 2.2.2. Sugar Fermentation Assay

The medium was prepared according to the China National Standard GB 4789.35-2016, and it included 5 g/L beef extract powder, 5 g/L tryptone, 5 g/L yeast extract, 0.5 mL/L Tween-80, 1.5 g/L agar powder, 1.4 g/L 1.6% (*w*/*v*) bromocresol violet in ethanol, and 5 g/L required sugar. Bacteria were washed three times with sterile saline (9 g/L NaCl) and resuspended in sterile water. Suspensions were adjusted to 10^9^ colony-forming units (CFU)/mL using turbidimetry, then inoculated at 1% (*v*/*v*) into sugar fermentation tubes containing smaller tubes. Colour changes and bubble formation were then observed.

### 2.3. Safety Evaluations

#### 2.3.1. Arginine Ammonia

The arginine broth solid medium was prepared as described previously [38], including 5 g/L tryptone, 5 g/L yeast extract, 0.5 g/L glucose, 2 g/L K_2_HPO_4_, 3 g/L L-arginine, and 15 g/L agar powder. An arginine-free broth medium was used as the control. The activated *L. brevis* strains were inoculated into two of the arginine broth solid mediums and incubated for 72 h at 37 °C. The production of a yellow precipitate from the drip addition of Nessler’s reagent showed a positive arginine ammonia production test result.

#### 2.3.2. Bile Salt Hydrolase

As previously described [39,40], bacteria were inoculated onto the MRS agar medium containing 5 g/L taurodeoxycholic acid (Sigma-Aldrich, Saint Louis, MO, USA) and incubated at 37 °C for 5 d. The taurodeoxycholic acid was hydrolysed by bile salt hydrolase into deoxycholic acid precipitates, the size of which reflected the enzyme activity.

#### 2.3.3. Haemolysis

Assays were performed as previously described [36]. Briefly, bacteria were spread on blood agar base medium (Bikeman, Changde, China) plates and incubated at 37 °C for 48 h. The non-haemolytic strains (γ-haemolytic) showed no effect or no haemolysis on these plates.

#### 2.3.4. Antibiotic Susceptibility

E-tests were used to determine the minimum inhibitory concentrations (MICs) for each antimicrobial agent [41]. Fifty microlitres of each bacterial suspension described above were spread onto MRS agar plates. E-test strips (Liofilchem, Roseto degli Abruzzi, Italy) were placed on the plate surface and incubated at 37 °C for 20 h.

### 2.4. In Vitro Simulations of Tolerance

#### 2.4.1. Gastric Acid

Assays were performed as previously described [41,42] with slight modifications. Bacteria were inoculated at 1% (*v*/*v*) into the MRS medium at pH 2.0 (simulating fasting), 2.5, and 3.0 (simulating satiety), followed by incubation at 37 °C and 100 rpm with shaking for 0–4 h. Samples were then collected hourly and counted on MRS agar plates using the dilution spread plate method, with incubation at 37 °C for 48 h. Survival was calculated using the following formula:

Survival rate = log_10_ N_t_/log_10_ N_0_ × 100% (1)

where N_t_ is the number of viable bacteria after incubation and N_0_ is the initial number of viable bacteria.

#### 2.4.2. Bile Salt

The MRS medium containing 3 g/L bile salt No. 3 (Ourchem, Shanghai, China) was prepared, and its pH was adjusted to 6.8 with hydrochloric acid. Bacterial suspensions were inoculated at 1% (*v*/*v*) into the MRS medium, followed by incubation at 37 °C and 100 rpm shaking for 0–8 h. Samples were collected at 2 h intervals and inoculated on MRS agar plates, followed by incubation at 37 °C for 48 h for cell counts.

#### 2.4.3. Simulated Human Gastric Juice

Human dietary suspensions were prepared as previously described [41,43] to simulate human gastric juice composed of 2 g/L NaCl and 3 g/L pepsin (SCR, Shanghai, China), and adjusted to pH 3.0 with hydrochloric acid. Bacteria were inoculated into 1% (*v*/*v*) of this preparation and incubated at 37 °C and 100 rpm with shaking for 0–4 h. Samples were collected at 1 h intervals for cell counts.

#### 2.4.4. Simulated Human Intestinal Fluid

Human dietary suspensions were mixed with 12.5 g/L NaHCO_3_, 3 g/L bile salt No. 3 (Ourchem, Shanghai, China), and 0.1% trypsin (Solarbio, Beijing, China), with pH adjusted to 6.8 with hydrochloric acid to mimic human intestinal fluid. Bacteria were inoculated into 1% (*v*/*v*) of this preparation and incubated at 37 ℃ and 100 rpm for 0–8 h. Samples were collected at 2 h intervals for cell counts.

### 2.5. Litchi Juice Fermentation

Fermentation was performed as previously described [44,45] with the following changes. Fresh ripe litchi fruits were washed with water, peeled, pitted, and homogenised using a juicer (Midea Group, Guangzhou, China). The pulp was passed through 8 layers of clean gauze and an 80-mesh stainless-steel sieve. The filtrate was clarified at 6950× *g* for 10 min at 4 °C, and the supernatant (litchi juice) was stored at −20 °C. A mixture of 20% litchi juice, 0.5% yeast extract, 0.5% monosodium glutamate (MSG), and 79% ultrapure water (all *w*/*w*) was sterilised at 95 °C for 15 min and then cooled to 35–40 °C. Bacteria were inoculated into the mixture at 10^7^ CFU/mL and incubated for 48 h at 37 °C. Samples were collected at 8 h intervals for analysis.

### 2.6. Acidity Measurements

Sample pH was determined using a digital meter (Mettler Toledo, FE28, Columbus, OH, USA). Titratable acidity was assayed using the China National Standard GB 12456-2021 and expressed as the percentage of lactic acid. A 5 mL sample was added to 10 mL of pure water, followed by the addition of 50 μL phenolphthalein indicator (5 g/L). The mixture was titrated with 0.1 M sodium hydroxide until the red colour did not fade within 30 s, and the volume of the sodium hydroxide standard solution was recorded. Titratable acidity was calculated using the following formula:

Titratable acidity = V_NaOH_ × c × 0.090/V_s_ × 100% (2)

where V_NaOH_ is the volume of sodium hydroxide solution consumed during titration in mL; c is the concentration of the sodium hydroxide standard solution in mol/L; and V_s_ is the volume of sample drawn in mL.

### 2.7. Bacterial Count

Viability was assessed according to the national standard GB 4789.35-2016 and previously reported methods [46] with slight modifications. The fermented juice was evenly mixed, and 0.6 mL was added to 5.4 mL of pure water, followed by serial 10-fold dilutions. Fifty microlitres of each dilution were then spread onto the MRS agar medium, followed by incubation at 37 °C for 48 h. The cell counts were expressed as log_10_ CFU/mL.

### 2.8. Determination of Antioxidant Capacity

#### 2.8.1. DPPH Radical Scavenging

The 2,2-diphenyl-1-picrylhydrazyl (DPPH) radical scavenging activity of fermented litchi juice was determined as previously described [41,47,48] with some modifications. Briefly, each sample was diluted in purified water to a final concentration of 20% (*v*/*v*), and 2.0 mL of the diluted sample was mixed with 0.2 mM DPPH (Tokyo Chemical Industry Co., Ltd., Tokyo, Japan) in ethanol (2.0 mL), with deionised water in place of the sample used as the control, and different concentrations of 2,6-di-tert-butyl-4-methylphenol (BHT; Shanghai Macklin Biochemical, Shanghai, China) were used instead of the sample as a positive control [49]. The mixture was incubated at 25 °C for 30 min in the dark, immediately followed by centrifugation at 6950× *g* at 4 °C for 10 min. The absorbance of the supernatant was immediately measured using a UV spectrophotometer (UV-1800; Shimadzu, Kyoto, Japan) at 517 nm. DPPH radical scavenging rates were calculated according to the following formula:

DPPH radical scavenging rate = (A_0_ − A_t_)/A_0_ × 100% (3)

where A_t_ is the absorbance value after treatment of the sample, and A_0_ is the absorbance value after treatment of the control group.

#### 2.8.2. Ferric Ion Reduction

The ferric ion-reducing antioxidant power (FRAP) was determined according to a previously reported method [50] with modifications. In total, 100 mL of 300 mM NaAc (pH 3.6), 10 mL of 10 mM tripyridyltriazine (dissolved in 40 mM HCl), and 10 mL of 20 mM FeCl_3_ were mixed to prepare the working solution. The fermented juice supernatant (0.2 mL) was mixed with the working solution (4.8 mL) and subjected to a water bath at 37 °C for 5 min. The absorbance value of the supernatant was measured at 593 nm using the UV spectrophotometer. Different concentrations of BHT were used instead of the sample for the positive control, and 0–1.6 mM FeSO_4_•7H_2_O was used as the standard solution. The standard curve was y (absorbance) = 0.9426x (FeSO_4_•7H_2_O equivalents) + 0.0151 (R^2^ = 0.9999). The FRAP was calculated using the Fe^2+^ concentration.

#### 2.8.3. ABTS Radical Scavenging

The ability to scavenge 2,2′-amino-di(3-ethyl-benzothiazoline sulphonic acid-6) ammonium salt (ABTS) radicals was determined according to Qi et al. [51] with modifications. An ethanol solution containing 7.29 mM ABTS and 2.545 mM K_2_S_2_O_8_ was placed in the dark at 25 °C for 12 h and used as the mother liquor. An appropriate amount of mother liquor was diluted to an absorbance value of 0.68–0.72 (734 nm) and used as the working solution. The fermented juice supernatant was diluted in pure water to a final concentration of 25% (*v*/*v*), and then 0.4 mL of the dilution was mixed with the working solution (3.6 mL). Purified water replaced the sample as a control, and different concentrations of BHT were used instead of the sample as the positive controls. The mixture was incubated at 25 °C in the dark for 5 min, and the absorbance of the supernatant was measured at 734 nm using UV spectrophotometry. The ABTS radical scavenging rate of the diluted sample was calculated using the following formula:

ABTS radical scavenging rate = (A_0_ − A_s_)/A_0_ × 100% (4)

where A_s_ is absorbance after treatment, and A_0_ is absorbance after treatment with the control.

### 2.9. Polyphenol

The total polyphenol content in the fermented mixed litchi juice was determined by folinphenol spectrophotometry as previously described [52] with some modifications. Briefly, the supernatant was diluted in pure water to a final concentration of 25% (*v*/*v*), and 0.6 mL was mixed with 150 g/L Na_2_CO_3_ solution (0.6 mL) and 1.5 mL folinphenol reagent (Shanghai Macklin Biochemical, Shanghai, China). The mixture was supplemented with pure water to a total volume of 6 mL. Absorbance of the mixture was measured using a UV spectrophotometer (778 nm) after incubation in a water bath at 40 °C in the dark for 1 h. With 0–60 mg/L gallic acid as the standard solution, the standard curve was y (absorbance) = 0.0191x (gallic acid equivalents content) + 0.011 (R^2^ = 0.9997). The total polyphenol content was calculated using the gallic acid concentration.

### 2.10. Flavonoids

The total flavonoid content was determined as previously described [53] with modifications. The fermented juice supernatant (0.5 mL) was mixed with 5% (*w*/*v*) NaNO_2_ (0.15 mL) and allowed to stand for 6 min, then 0.15 mL 10% (*w*/*v*) AlCl_3_•6H_2_O was added and mixed. The mixture was allowed to stand for 6 min, and 4% (*w*/*v*) NaOH (2.0 mL) was added and supplemented to 3 mL with pure water. After the mixture was left to stand for 5 min, the absorbance was determined using a UV spectrophotometer (510 nm). For the standard solution, 0–400 mg/L rutin was used, the standard curve was y (absorbance) = 0.0022x (rutin equivalents content) − 0.0148 (R^2^ = 0.9997), and the total flavonoid content was calculated using the rutin concentration.

### 2.11. Acetic Acid

Acetic acid was analysed using an Essentia LC-20A HPLC system (Shimadzu, Kyoto, Japan) equipped with an auto-sampler, binary pump, and a differential refraction detector. Filtration was performed using a 0.45 μm filter membrane before injection. Ten microlitres of the sample was injected into an Aminex HPX-87H column (300 mm× 7.8 mm) and eluted using 0.05% (*w*/*v*) aqueous H_2_SO_4_ solution at a flow rate of 0.5 mL/min and a column temperature of 60 °C (R^2^ = 0.9992).

### 2.12. Statistical Analysis

Data were analysed using OriginLab 9.0 software (OriginLab Co., Northampton, MA, USA) and SPSS (version 25, IBM Corporation, Armonk, New York, NY, USA). Data are presented as the means ± standard deviation (SD). Experiments were repeated three times (*n* = 3). Significant differences (*p* ˂ 0.05) were identified using Tukey’s honestly significant difference test. The letters (a, b, c, d) indicate significantly different means at *p* < 0.05 based on one-way analyses of variance. Correlations were analysed using the Pearson method.

## 3. Results

### 3.1. Strain Isolation and Identification

**Figure 1:**
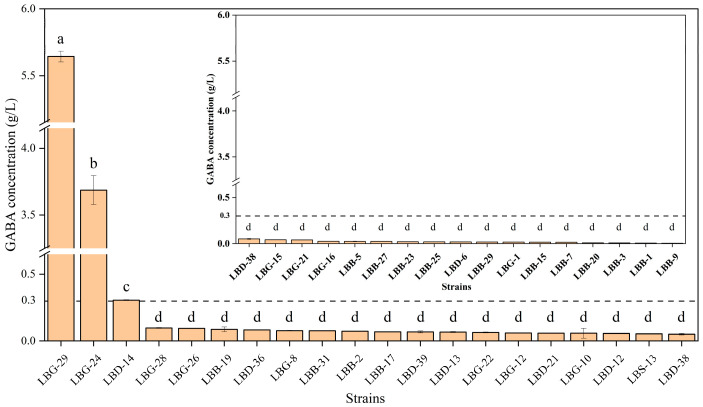
Production of GABA (g/L; *n* = 3) by the isolated lactic acid bacteria strains. Values are the means ± SD. Different letters (a, b, c, d) indicate means that are significantly different at *p* < 0.05.

Fifty LAB isolates were obtained from the four traditional Chinese pickles, and they were typically catalase-negative and Gram-positive. HPLC showed that 36 of the strains produced GABA, 3 of which were classified as producing high levels (>300 mg/L) [54], with LBG-29 being the highest (5.64 ± 0.04 g/L) (Figure 1), followed by LBG-24 (3.69 ± 0.11 g/L) and LBD–14f (0.31 ± 0.01 g/L). The three strains used in subsequent assays (LBG-29, LBG-24, and LBD–14) were *L. brevis*, as determined by 16S rRNA sequencing (Table 1).

### 3.2. Physio-Biochemical Analysis

LBG-29, LBG-24, and LBD–14 were negative for methyl red, indole, and V-P. They did not hydrolyse hippurate or starch, exhibited no gelatinase liquefaction enzyme or urease, and did not reduce nitrate or produce hydrogen sulphide (Table 2). LBG-29 hydrolysed esculin to aesculetin, but LBG-24 and LBD–14 did not. The ability to utilise different saccharides was evaluated using the sugar fermentation assay, and none of the strains produced gas (Table 3).

### 3.3. In Vitro Safety Evaluations

To evaluate their safety in vitro, LBG-29, LBG-24, and LBD–14 were each assessed for arginine ammonia production, bile salt hydrolase activity, haemolysis, and drug susceptibility. None of the strains decomposed arginine to produce ammonia or had bile salt hydrolase activity (Table 4). Furthermore, all three strains were γ-haemolytic (Table 4) and did not lyse erythrocytes. They were also all resistant to vancomycin and ciprofloxacin, and had varying degrees of resistance to erythromycin, tetracycline, gentamicin, and ampicillin (Table 5).

### 3.4. Gastric Acid and Bile Acid Tolerance

**Figure 2:**
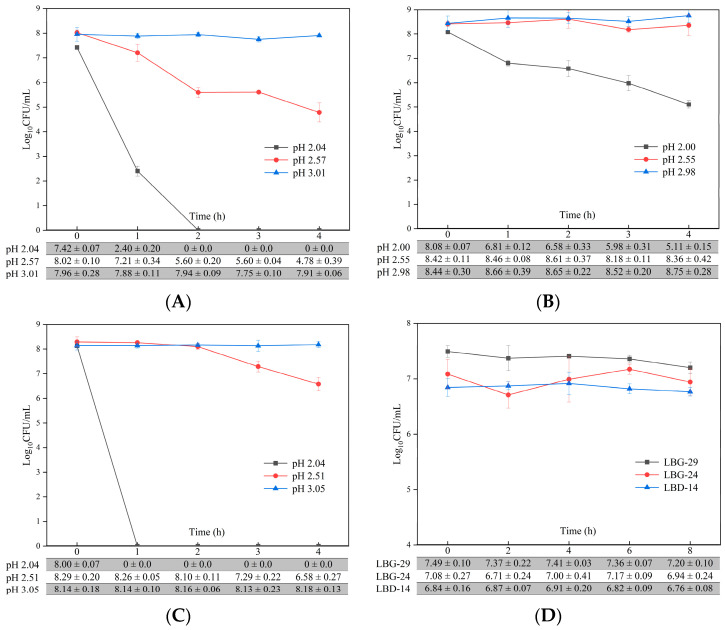
Tolerance of the three *Levilactobacillus brevis* strains to simulated gastric acid and bile salts in vitro. (**A**–**C**) Tolerance of LBG-29 (**A**), LBG-24 (**B**), and LBD–14 (**C**) to simulated gastric acid in vitro. (**D**) Tolerance of LBG-29, LBG-24, and LBD–14 to simulated intestinal bile salts (3 g/L) in vitro. Values are means ± SD.

The viability of LBG-24 significantly decreased after 4 h at pH 2.0 (63%) (Figure 2A–C), while LBG-29 and LBD–14 were not detected after 2 h and 1 h at pH 2.0, respectively. Tolerance to simulated gastric juice increased at pH 2.5, compared with 0 h, and the survival rates of LBG-29 and LBD–14 were significant at 60% and 79%, while that of LBG-24 was not significant at 99%, after 4 h of exposure. Compared with 0 h, viability did not change at pH 3.0 and the survival rates for LBG-29, LBG-24, and LBD–14 were not significant at 99%, 104%, and 100%, respectively, after 4 h. The viability of LBG-29 slightly decreased after 8 h in the simulated intestinal fluid containing 3 g/L bile salts; however, the order of magnitude remained unchanged, and the survival rate was significant at 96% (Figure 2D), while the survival rates of LBG-24 and LBD–14 were not significant at 98% and 99%, respectively.

### 3.5. Simulated Human Gastric Juice and Intestinal Fluid Tolerance

**Figure 3:**
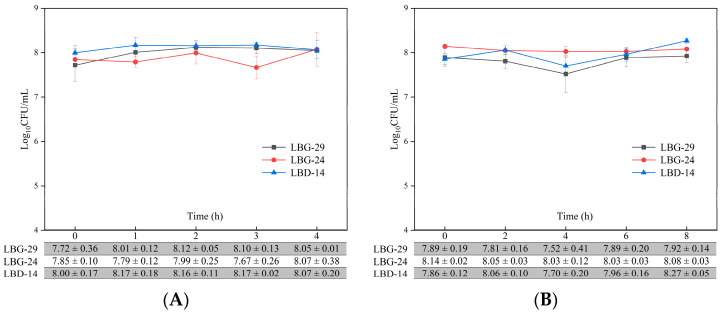
Tolerance of the three *Levilactobacillus brevis* strains to human gastric juice (**A**) and intestinal fluid (**B**) under simulated satiety in vitro. Values are means ± SD.

A slight change in viability in the simulated human gastric juice was observed under satiety conditions within 4 h, but viability after 4 h was not significantly different from that at the beginning; the final survival rates of LBG-29, LBG-24, and LBD–14 were 104%, 103%, and 101%, respectively (Figure 3A). Furthermore, the survival rates after 8 h of incubation in the simulated human intestinal fluid were 101%, 99%, and 105%, respectively (Figure 3B).

### 3.6. pH and Acidity during Fermentation

**Figure 4:**
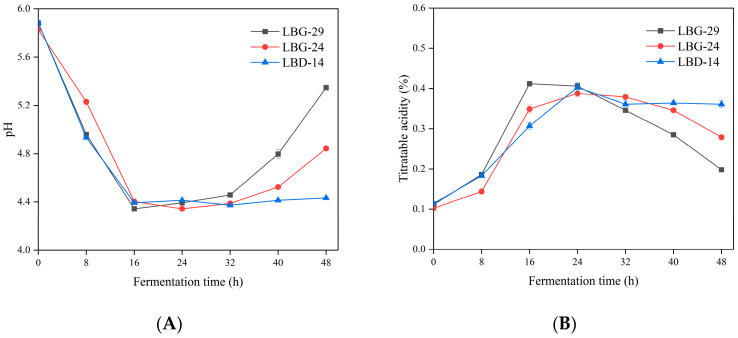

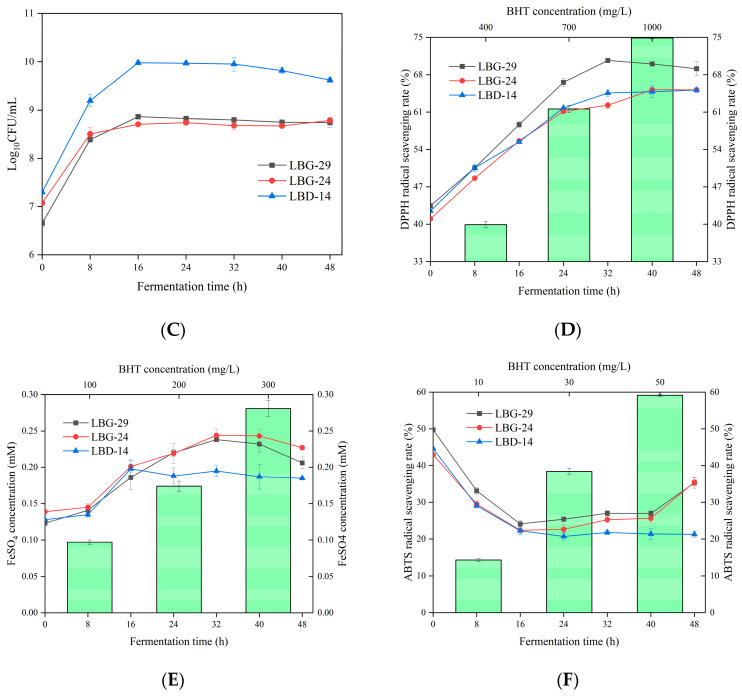
Fermentation time dependence of pH (**A**), titratable acidity (**B**), viability (**C**), DPPH radical scavenging (**D**), FRAP (**E**), and ABTS radical scavenging (**F**) in litchi juice fermentation. Values are means ± SD.

The fermentation pH with LBG-29, LBG-24, and LBD–14 was significantly decreased after 48 h of fermentation (Figure 4A). The pH of the LBG-29 fermentation significantly decreased during the initial 16 h, reached its lowest value of 4.34 after 16 h, and then gradually but significantly increased from 16 to 48 h. The pH value of the LBG-24 fermentation showed the same trend as that of the LBG-29 as it decreased and then significantly increased, while that of the LBG-24 fermentation reached its lowest pH of 4.34 at 24 h. The LBD–14 pH significantly decreased from 5.89 to 4.39 during the initial 16 h of fermentation and slightly but significantly fluctuated after 16 h but was always within the range of 4.37–4.44.

The titratable acidity for all three strains significantly increased after 48 h of fermentation. The acidity of LBG-29 significantly increased to a maximum of 0.41% during the initial 16 h, remained stable from 16 to 24 h, and gradually but significantly decreased from 24 to 48 h. The acidity of the LBG-24 significantly increased to its highest level of 0.39% during the initial 24 h, remained stable from 24 to 32 h, and gradually but significantly decreased from 32 to 48 h. The acidity of the LBD–14 significantly increased during the initial 24 h, slightly but significantly decreased from 24 to 32 h, and stabilised after 32 h (Figure 4B). There was a significant negative correlation (r ≤ −0.628) between pH and titratable acidity.

### 3.7. Viability during Fermentation

The viability of the *L. brevis* strains LBG-29, LBG-24, and LBD–14 significantly increased during the initial 16 h of fermentation (Figure 4C). Furthermore, the LBG-29 and LBG-24 remained stable at 8 log_10_ CFU/mL from 16 to 48 h. The viability with LBD–14 remained in the same order of 9 log_10_ CFU/mL of magnitude from 16 to 40 h and significantly decreased from 40 to 48 h.

### 3.8. Antioxidative Capacity

DPPH is widely used to evaluate free radical scavenging activity. The use of the *L. brevis* strains for litchi juice fermentation significantly enhanced the radical scavenging ability of the DPPH (Figure 4D). After fermentation for 48 h, the DPPH radical scavenging ability of the LBG-29 fermentation was the strongest, and the radical scavenging rate of the 20% fermented litchi juice increased from 43.46% to 69.13%. The scavenging activity of LBG-29 and LBD–14 significantly increased during the initial 32 h of fermentation, and the DPPH radical scavenging rate of 20% LBD-14-fermented litchi juice reached 64.58% and remained stable from 32 to 48 h. The DPPH radical scavenging of the LBG-24 significantly increased over the initial 40 h, and the DPPH radical scavenging rate of the 20% LBG-24-fermented litchi juice reached 65.20% and remained stable from 40 to 48 h. After fermentation for 48 h, the DPPH radical scavenging ability of the three 20%-fermented litchi juices was stronger than that of the 700 mg/L BHT.

Similarly, the FRAP significantly increased by 49.19%–67.48% after fermentation with the LBG-29, LBG-24, and LBD–14 (Figure 4E), and was stronger than that of the 200 mg/L BHT. During the initial 8 h, the FRAP of the LBG-29 and LBD–14 remained stable, while that of the LBG-24 increased significantly. The FRAP of the LBG-29 and LBG-24 increased significantly and continuously from 8 to 32 h, remained stable from 32 to 40 h, and decreased slightly but significantly from 40 to 48 h. However, the FRAP of the LBD-14 increased continuously and significantly from 8 to 16 h and remained stable from 16 to 48 h.

In contrast with the overall increases for both DPPH radical scavenging and FRAP after fermentation, the ABTS radical scavenging decreased significantly (Figure 4F). The ABTS radical scavenging of LBG-29, LBG-24, and LBD–14 decreased significantly during the initial 16 h of fermentation, after which, the rates for the 25%-fermented litchi juices were 24.11%, 22.38%, and 22.26%, respectively. The scavenging of the LBD–14 was stable from 16 to 48 h. ABTS radical clearance by LBG-29 and LBG-24 increased slightly but not significantly from 16 to 40 h and then increased significantly from 40 to 48 h. After fermentation, the ABTS radical scavenging ability of the three 25%-fermented litchi juices was stronger than that of the 30 mg/L BHT. After 48 h fermentation, the ABTS radical scavenging rates of the 25%-fermented LBG-29, LBG-24, and LBD-14 litchi juices were 35.30%, 35.50%, and 21.27%, respectively. There was a significant positive correlation (r ≥ 0.830) between the changes in DPPH radical scavenging activity and FRAP during fermentation for each of the three strains.

### 3.9. Polyphenol Production during Fermentation

**Figure 5:**
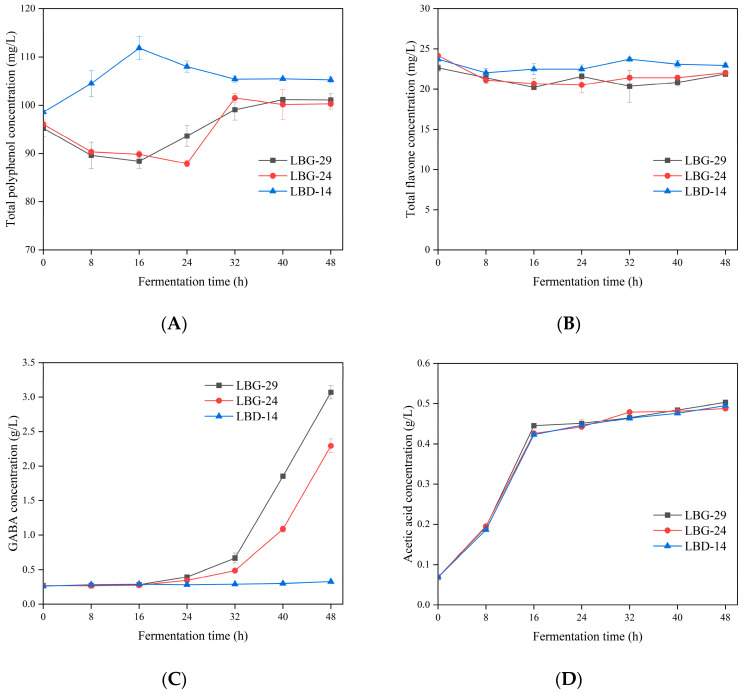
Fermentation time dependence of the total polyphenol (**A**), total flavonoid (**B**), GABA (**C**), and acetic acid (**D**) contents. Values are the means ± SD.

The total polyphenol content of the mixed lychee juice at 48 h after fermentation slightly but significantly increased, with a growth rate of 4.42%–6.80% (Figure 5A). The total polyphenol content of the LBG-29-fermented litchi juice significantly decreased by 7.18% during the initial 16 h, increased significantly from 16 to 32 h, and remained stable from 32 to 48 h. The polyphenol content of the LBG-24 decreased significantly by 8.51% over the initial 24 h, increased significantly from 24 to 32 h, and remained stable from 32 to 48 h. In contrast with LBG-29 and LBG-24, the LBD–14 increased significantly during the initial 16 h, decreased significantly from 16 to 32 h, and remained stable from 32 to 48 h. According to the Pearson correlation coefficient analysis, the changes in total polyphenol content during fermentation were positively correlated with the changes in DPPH (except LBG-24) and the FRAP (r ≥ 0.455).

### 3.10. Flavonoid Production during Fermentation

The total flavonoid content fluctuated during fermentation, but overall, it decreased during the initial 48 h (Figure 5B). After fermentation, the total flavonoid content of the LBG-29, LBG-24, and LBD-14 was reduced by 0.76, 2.12, and 0.76 mg/L, with reduction rates of 9.36%, 3.15%, and 4.36%, respectively. The total flavonoid content of the LBG-29 and LBG-24 fermentations were found to be significantly positively correlated with ABTS (r ≥ 0.665).

### 3.11. GABA

After fermentation for 48 h, the highest GABA content was found in the LBG-29-fermented litchi juice (Figure 5C). LBG-29 produced an insignificant amount of GABA during the initial 16 h of fermentation but produced a significant amount from 16 to 48 h with a maximum concentration of 3.07 ± 0.10 g/L at 48 h. LBG-24 produced an insignificant amount of GABA during the initial 24 h but produced a significant amount from 24 to 48 h, with a maximum concentration of 2.29 ± 0.10 g/L at 48 h. However, fermentation by the LBD–14 produced a maximum of 0.327 ± 0.01 g/L GABA.

### 3.12. Acetic Acid

Acetic acid increased significantly by 0.35–0.38 g/L during the initial 16 h of fermentation (Figure 5D), but increased by ≤ 0.08 g/L from 16 to 48 h. After fermentation, the acetic acid contents of the litchi juice fermented using LBG-29, LBG-24, and LBD-14 were 0.50 ± 0.01 g/L, 0.49 ± 0.01 g/L, and 0.50 ± 0.01 g/L, respectively.

## 4. Discussion

To meet the demands of the rapidly growing probiotic market, new probiotics with multiple functions must be screened and identified. A strain’s functionality, safety, and tolerance to the human gastrointestinal tract are important criteria to consider for potential probiotics. In this study, *L. brevis* strains LBG-29, LBG-24, and LBD-14 were screened from traditional Chinese pickles based on their ability to synthesise high levels of GABA. LAB, including *L. plantarum*, *L. brevis*, and *Pediococcus pentosaceus,* often produce GABA. However, their yields vary greatly, and *L. brevis* is generally the dominant GABA-producing species [55]. The strains LBG-29, LBG-24, and LBD-14, which were identified in this study, do not catabolise arginine to produce ammonia or hydrolysate bile salts, and are not hemolytic. These characteristics indicate that they are safe for humans and will not show in vivo toxicity caused by the intestinal absorption of amino acid decarboxylation metabolites [56], nor will there be the harmful intestinal effects that result from increased bile salt degradation, such as colon cancer, mucosal inflammation, and gallstone formation [57]. Drug susceptibility is also an important aspect of strain safety, as resistance genes can be transferred to other microorganisms (including gut pathogens) if they are located on mobile genetic elements [58]. The sensitivity of lactobacilli to antibiotics has obvious inter-species dependence. They may have a natural resistance to some antibiotics; however, vancomycin resistance is usually considered an intrinsic property [59,60].

In addition to safety, tolerance to low pH and high bile salt concentrations are crucial properties of probiotics [61]. LBG-29, LBG-24, and LBD-14 exhibited low tolerance to a pH 2.0 environment and high tolerance to a pH 3.0 environment. Similarly, *L. brevis* DSM 32386, isolated from traditional alpine cheese, was found to have a survival rate of 82–103% after 3 h at pH 3.2 but was not viable after 3 h at pH 2.0 [62]. A low pH (2.0) environment thus results in undissociated HCl around the cell membrane, which easily enters the cytoplasm. This acidification eventually causes protein denaturation and DNA damage [63]. This may be the reason that the *L. brevis* strains could not survive at a low pH. At the same time, bacteria have multiple physiological mechanisms with which to tolerate an acidic environment, including ATPase activation and cell-envelope remodelling. This may explain why each of our three strains was viable after 4 h in simulated gastric juice at pH 3.0. Bile salts are essential for balancing gut microbiota and carbohydrate metabolism in the small intestine, and their average concentration is 0.3% (*w*/*v*) [64]. The three strains identified in this study did not have bile salt hydrolase activity. Their tolerance to high bile salt concentrations may be attributed to changes in the composition of the bacterial proteins and lipids on the cell membrane, leading to decreased bile salt diffusion. Alternatively, it may be caused by the active efflux pumps and transporters that are ubiquitous in Gram-positive bacteria and protect cells from toxic substances [63,65]. Pepsin catalyses protein digestion, and its production is stimulated by the release of hydrochloric acid from the stomach. At the same time, trypsin production by the pancreas promotes lipid metabolism in humans [62]. Therefore, pepsin and trypsin are better at simulating the composition of human gastric and intestinal juices [66]. Similarly, LBG-29, LBG-24, and LBD-14 also exhibited tolerance to human gastrointestinal fluids under satiety conditions in vivo.

To provide sufficient health benefits to the host, the number of viable probiotic bacteria after fermentation in functional foods must meet the defined requirements (≥10^6^ CFU/mL) [67]. The results indicated that LBG-29, LBG-24, and LBD-14 all met the requirements for viable bacteria during fermentation, and consequently, they were identified as potential starter cultures for litchi juice fermentation. The various physical and chemical components and functional properties of fermented foods will change with the growth and metabolism of strains during fermentation [68,69]. The effects of *L. brevis* on fermentation were evaluated by measuring pH, total acids, antioxidant properties, and functional active ingredients. During fermentation, a significant negative correlation was found between pH and titratable acidity. This pH decrease may be related to the production of lactic and organic acids during bacterial growth, while the increase in pH may be related to GABA production, as glutamate decarboxylation in LAB and the transport of GABA via the antiporter cause hydrogen ion depletion and GABA production, leading to increased cytoplasmic and environmental alkalinity [70]. A significant positive correlation was identified between DPPH radical scavenging activity and the FRAP in fermented foods during fermentation, while LBG-29 and LBG-24 exhibited different trends for ABTS radical scavenging activity, which may be due to different antioxidant mechanisms. Both DPPH antioxidant activity and the FRAP are related to sequential proton-loss electron transfer, while the ABTS radical scavenging reaction is a hydrogen atom transfer pathway [71]. In addition, the increased antioxidant activity may be related to the improved utilisation of polyphenols with proton transfer properties in LAB fermentation [72]. It has been reported that the generation and degradation of antioxidants and the synergy between bioactive compounds during LAB fermentation significantly contribute to the changes in DPPH, FRAP, and ABTS [73]. Many studies have found that the use of LAB strains to ferment foods can improve polyphenol and flavonoid contents in the fermentation matrix, thereby improving their functional properties, such as their antioxidant activities [48,74]. It is thus crucial to explore the changes in total polyphenols and flavonoids during the fermentation of litchi juice by *L. brevis*.

In this study, the changes in total polyphenol content during fermentation were found to be positively correlated with the changes in the DPPH (except LBG-24) and FRAP. The increased polyphenol content may be an important reason for the enhanced antioxidant activity. The variations in polyphenol content are related to the degradation and formation of some polyphenolic compounds by LAB, while the antioxidant activity of polyphenolic compounds varies owing to the location and number of hydroxyl groups [75]. For example, Li et al. [76] found that DPPH in LAB-fermented Muzao juice was significantly positively correlated with the phenolic calcium and cinnamic acids and was significantly negatively correlated with chlorogenic acid. Therefore, the transformation, formation, and degradation of phenolic compounds with oxidative activity are all factors that contribute to the changes in the antioxidant activities during fermentation. Cele et al. [73] also reported that the fermentation of mango juice by LAB increased the total polyphenols, and this was consistent with our findings. During the fermentation of LBG-29 and LBG-24, the total polyphenol content decreased significantly in the early stages of fermentation, and this may be due to the low number of viable cells for this strain at this stage and the slow oxidative decomposition of phenolic compounds into quinones owing to the presence of oxygen. Subsequently, the number of viable cells increased significantly, and a large number of β-glucosidase and phenolic esterases produced by the strains hydrolysed phenolic compounds to form a variety of free phenolic substances, which eventually led to a significant increase in the total polyphenol content [77]. In contrast, the total flavonoid content decreased after fermentation, which was consistent with the results of Malik et al. [78], who found that the fermentation of beetroot juice and carrot juice with lactic acid bacteria led to a decrease in the total flavonoid content. *L. brevis* fermentation is clearly related to a decrease in total flavonoid content, and the ability of a strain to produce hydrolytic enzymes may be responsible for this process [77]. The total flavonoid content of the LBG-29 and LBG-24 fermentations was significantly positively correlated with ABTS. Therefore, the decrease in flavonoids may be the cause for the decrease in ABTS radical scavenging activity.

Many studies have shown that suitable probiotics (especially LAB) significantly increase the GABA content of fermented juices. One of the key goals of this study was to obtain GABA from the fermentation process. As expected, the LBG-29 and LBG-24 produced large amounts of GABA after fermentation. A substantial GABA synthesis was accompanied by a marked increase in extracellular pH for LBG-29 and LBG-24, and GABA production showed a significant lag with respect to bacterial growth. Similarly, GABA was only detected in blueberry juice (supplemented with yeast extract and sodium glutamate) after 48 h of fermentation with *L. brevis* CRL 2013 [44]. This may be because the growth and metabolism of *L. brevis* cause a gradual reduction in environmental pH, which provides suitable conditions for the function of glutamate decarboxylase. Overall, the results suggest that LBG-29 and LBG-24 are suitable probiotic starter cultures for litchi juice fermentation. A significant increase in acetate content after fermentation in *L. brevis* was also found, which was surprising, as acetic acid is an important short-chain fatty acid that plays a crucial role as a substrate for cholesterol and lipid metabolism. In addition, it is also relevant to human gut homeostasis owing to its association with diet, gut microbiota, and host energy metabolism [79,80]. In conclusion, the results suggest that litchi juice fermented using LBG-29 and LBG-24 will have multiple benefits in vivo, such as increasing active substances, providing probiotics, and improving health functions.

## 5. Conclusions

In this study, three *L. brevis* strains (LBG-29, LBG-24, LBD-14) that produce high levels of GABA (>300 mg/L) were isolated from traditional Chinese pickles. The three strains could all survive in simulated human gastric juice and intestinal fluid under satiety conditions. They were determined to be suitable starter cultures for litchi juice fermentation, as the juice facilitated significant increases in viable bacteria. The *L. brevis* fermentation also significantly increased the antioxidant capacity (DPPH and FRAP) and total polyphenol content of the litchi juice. The increase in antioxidant activity may be related to the increase in total polyphenol content. Furthermore, LBG-29 and LBG-24 fermentation significantly increased the levels of GABA and short-chain fatty acids (acetic acid) in the litchi juice. Based on the above findings, the LBG-29 and LBG-24 strains were identified as suitable for use in the development of functional juice drinks; however, the functional benefits of these juices on human health and product shelf life will require further investigation. Overall, the results of this study will provide new directions and ideas for the development of healthy foods with multiple functional activities, improve consumer choice and the economics of fruit utilisation, and inspire industrial ideas for fruit juice food processing enterprises.

## Figures and Tables

**Table 1:** Isolate identification.

Strain	Source	Blast Identification	Preservation Institution	Preservation Number	Accession Number
LBG-29	Pickled cucumber	*L. brevis*	CCTCC (China Center for Type Culture Collection)	M 2022686	ON287036
LBG-24	Pickled cucumber	*L. brevis*	-	-	ON287037
LBD–14	Pickled bell pepper	*L. brevis*	-	-	ON287034

**Table 2:** Physiological and biochemical characteristics of the screened *Levilactobacillus brevis* strains.

Assay	LBG-29	LBG-24	LBD–14
Carbohydrate metabolism			
Methyl red	−	−	−
V-P	−	−	−
Esculin hydrolysis	+	−	−
Hydrolysed hippurate	−	−	−
Starch hydrolysis	−	−	−
Sugar fermentation	The results are shown in Table 3		
Protein and amino acid metabolism			
Indole	−	−	−
Hydrogen sulphide	−	−	−
Urease production	−	−	−
Gelatin liquefaction	−	−	−
Enzymes			
Nitrate reduction	−	−	−

+, positive reaction; −, negative reaction.

**Table 3:** Sugar fermentation.

Sugar	LBG-29	LBG-24	LBD–14
Amygdalin	+	−	+
DL-Arabinose	+++	−	+++
D-(+)-Cellobiose	++	−	++
Dulcitol	+	−	+
Erythritol	+++	+++	+++
Esculin hydrate	++	−	++
D-Fructose	+++	+++	+++
Glucose	+++	+++	+++
Glycerine	+	−	+
Sodium hippurate	++	−	++
myo-Inositol	+	−	+
Inulin	+	−	+
Lactose	+	−	+
D-(+)-Maltose monohydrate	++	++	+++
D(+)-Mannose	++	−	++
D-(−)-Ribose	+++	+++	+++
L-(+)-Ribose	+++	−	+++
D-Sorbitol	+	−	+
Stachyose	++	−	++
D-Raffinose	+	−	+
Mannitol	+	−	+
D-(+)-Melezitose monohydrate	+	−	+
D-(+)-Melibiose monohydrate	+++	+++	+++
L-Rhamnose monohydrate	++	−	++
D(−)-Salicin	+	−	+
L(−)-Sorbose	++	−	++
Soluble starch	+	+	+
Sucrose	+	−	+
D-(+)-Trehalose anhydrous	+	−	+
D(+)-Xylose	+++	+++	+++
D-Gluconic acid sodium salt	+++	++	+++
Xylan	+++	+++	+++
Arbutin	+	−	+
Maltotriose	++	−	++
(+)-Arabinogalactan	+	−	+
Dextrin	+	−	+
Isomalto-oligosaccharide	+++	+++	+++
Fructo-oligosaccharide	+	−	+
Maltodextrin	++	−	++
Pectin	+++	−	+++

+++, positive reaction (completely yellow); ++, weak positive reaction (light yellow); +, weak positive reaction (light purple); −, negative reaction (deep purple).

**Table 4:** In vitro safety evaluations of the three *Levilactobacillus brevis* strains.

Strain Code	*Levilactobacillus brevis* LBG-29	*Levilactobacillus brevis* LBG-24	*Levilactobacillus brevis* LBD–14
Deamination of arginine	−	−	−
Bile salt hydrolase activity	−	−	−
Haemolysis	−	−	−

−, negative reaction.

**Table 5:** Antibiotic susceptibility and minimum inhibitory concentration of the three *Levilactobacillus brevis* strains.

Antibiotics	LBG-29	LBG-24	LBD–14
Vancomycin (0.016–256 mg/L)	>32 (+)	>32 (+)	>32 (+)
Erythromycin (0.016–256 mg/L)	1.0 (−)	0.19 (−)	0.50 (−)
Tetracycline (0.016–256 mg/L)	5 (−)	1.5 (−)	5 (−)
Gentamicin (0.016–256 mg/L)	6 (−)	5 (−)	5 (−)
Ampicillin (0.016–256 mg/L)	1.000 (−)	0.125 (−)	0.750 (−)
Ciprofloxacin (0.002–32 mg/L)	>32 (+)	>32 (+)	>32 (+)

+, resistant; −, sensitive.

## Data Availability

The data presented in this study are available on request from the corresponding author.

## Abbreviations

ABTS	2,2′-amino-di(3-ethyl-benzothiazoline sulphonic acid-6)ammonium salt
BHT	2,6-di-tert-butyl-4-methylphenol
CFU	colony-forming units
DPPH	2,2-diphenyl-1-picrylhydrazyl
FRAP	ferric ion-reducing antioxidant power
GABA	gamma-amino butyric acid
HPLC	high-performance liquid chromatography
LAB	lactic acid bacteria
*L. brevis*	*Levilactobacillus brevis*
MRS agar medium	de Man, Rogosa, and Sharpe agar medium
SD	standard deviation
